# The Social Component of Environmental Enrichment Is a Pro-neurogenic Stimulus in Adult c57BL6 Female Mice

**DOI:** 10.3389/fcell.2019.00062

**Published:** 2019-04-26

**Authors:** Elena P. Moreno-Jiménez, Jerónimo Jurado-Arjona, Jesús Ávila, María Llorens-Martín

**Affiliations:** ^1^Department of Molecular Neuropathology, Centro de Biología Molecular Severo Ochoa, CBMSO, CSIC-UAM, Madrid, Spain; ^2^Department of Molecular Biology, Faculty of Sciences, Universidad Autónoma de Madrid, Madrid, Spain; ^3^Center for Networked Biomedical Research on Neurodegenerative Diseases (CIBERNED), Madrid, Spain

**Keywords:** adult hippocampal neurogenesis, environmental enrichment, social enrichment, retrovirus, behavior

## Abstract

In rodents, the hippocampal dentate gyrus gives rise to newly generated dentate granule cells (DGCs) throughout life. This process, named adult hippocampal neurogenesis (AHN), converges in the functional integration of mature DGCs into the trisynaptic hippocampal circuit. Environmental enrichment (EE) is one of the most potent positive regulators of AHN. This paradigm includes the combination of three major stimulatory components, namely increased physical activity, constant cognitive stimulation, and higher social interaction. In this regard, the pro-neurogenic effects of physical activity and cognitive stimulation have been widely addressed in adult rodents. However, the pro-neurogenic potential of the social aspect of EE has been less explored to date. Here we tackled this question by specifically focusing on the effects of a prolonged period of social enrichment (SE) in adult female C57BL6 mice. To this end, 7-week-old mice were housed in groups of 12 per cage for 8 weeks. These mice were compared with others housed under control housing (2–3 mice per cage) or EE (12 mice per cage plus running wheels and toys) conditions during the same period. We analyzed the number and morphology of Doublecortin-expressing (DCX^+^) cells. Moreover, using RGB retroviruses that allowed the labeling of three populations of newborn DGCs of different ages in the same mouse, we performed morphometric, immunohistochemical, and behavioral determinations. Both SE and EE increased the number and maturation of DCX^+^ cells, and caused an increase in dendritic maturation in certain populations of newborn DGCs. Moreover, both manipulations increased exploratory behavior in the Social Interaction test. Unexpectedly, our data revealed the potent neurogenesis-stimulating potential of SE in the absence of any further cognitive stimulation or increase in physical activity. Given that an increase in physical activity is strongly discouraged under certain circumstances, our findings may be relevant in the context of enhancing AHN via physical activity-independent mechanisms.

## Introduction

Adult neurogenesis occurs in a limited number of brain regions in most mammalian species (Altman and Das, 1965; Kempermann et al., 2018; Moreno-Jiménez et al., 2019). Among these regions, the hippocampal dentate gyrus gives rise to newly generated dentate granule cells (DGCs) throughout life. This process, named adult hippocampal neurogenesis (AHN), converges in the functional integration of mature DGCs into the trisynaptic hippocampal circuit (Zhao et al., 2006). AHN is believed to participate in certain types of hippocampal-dependent learning, as well as in mood regulation (Sahay et al., 2011; Hill et al., 2015). In fact, this process is impaired in animal models of neurodegenerative and psychiatric diseases (Lazarov and Marr, 2010; Lazarov et al., 2010).

Adult hippocampal neurogenesis encompasses a series of sequential, tightly regulated stages (Zhao et al., 2006). Both intrinsic and extrinsic factors regulate the integration of newly generated neurons into the hippocampal circuitry. Among these factors, environmental enrichment (EE) and physical exercise are two of the most potent paradigms that stimulate AHN (Kempermann et al., 1997; van Praag et al., 1999). In fact, they exert neuroprotective actions both on healthy and diseased animals (van Praag et al., 2000, 2002; Teixeira et al., 2018). In contrast, social isolation causes stress for social rodents and markedly decreases the rate of AHN (Stranahan et al., 2006; Ibi et al., 2008).

Environmental enrichment generally consists of a combination of three major components, namely physical activity, cognitive stimulation, and social interaction. An increased level of physical activity is usually achieved by including voluntary running wheels in the enrichment cages. Cognitive stimulation is ensured by periodically changing the non-social components of the cage, such as toys, tubes or bedding material. Finally, the social component of EE, namely increased social interaction, occurs naturally as a consequence of the higher number of mice housed together in enriched cages. In this regard, the individual contribution of the cognitive and physical activity components of EE to the pro-neurogenic effects of this protocol during adulthood has been extensively addressed (van Praag et al., 1999; Steiner et al., 2008; Fabel et al., 2009). Previous studies demonstrate that physical activity and EE increase AHN via independent mechanisms (Olson et al., 2006; Garthe et al., 2016). However, the selective contribution of the social component of EE during adulthood has received less attention to date. In this regard, numerous studies have examined the long-term cellular and behavioral consequences of brief periods of EE applied early after weaning in comparison to social isolation (Branchi and Alleva, 2006; Branchi et al., 2006a,b, 2013; Curley et al., 2009; Gracceva et al., 2009; Cirulli et al., 2010; D’Andrea et al., 2010). However, to the best of our knowledge, the pro-neurogenic effects of a prolonged period of social enrichment (SE) during adulthood in comparison to EE and control housing conditions have not been examined to date. Here we addressed this question by determining the specific contribution of SE to the pro-neurogenic and behavioral effects of EE during adulthood in female C57BL6/J mice.

## Materials and Methods

### Animals

Five-week-old female C57BL6/J Ola Hsd mice were purchased from EnVigo laboratories (Spain). Animals were housed at the *Centro de Biología Molecular “Severo Ochoa”* (CBMSO) in a specific pathogen-free colony facility in accordance with European Community Guidelines (directive 86/609/EEC) and handled following European and local animal care protocols. Given that the hierarchy/dominance relationships established between male mice have a negative impact on AHN (Kozorovitskiy and Gould, 2004; McQuaid et al., 2018), only female mice were used in this work in all the housing conditions tested. Animals were left undisturbed for 2 weeks before starting any experimental manipulation. During this period, they were housed in groups of four mice per cage. Experiments were approved by the CBMSO Ethics Committee (AEEC-CBMSO-23/172) and the National Ethics Committee (PROEX 205/15). In stereotaxic injection experiments, five mice were used for each experimental condition. In cell count and behavioral determination experiments, seven animals per experimental condition were used.

### Experimental Design

**FIGURE 1:**
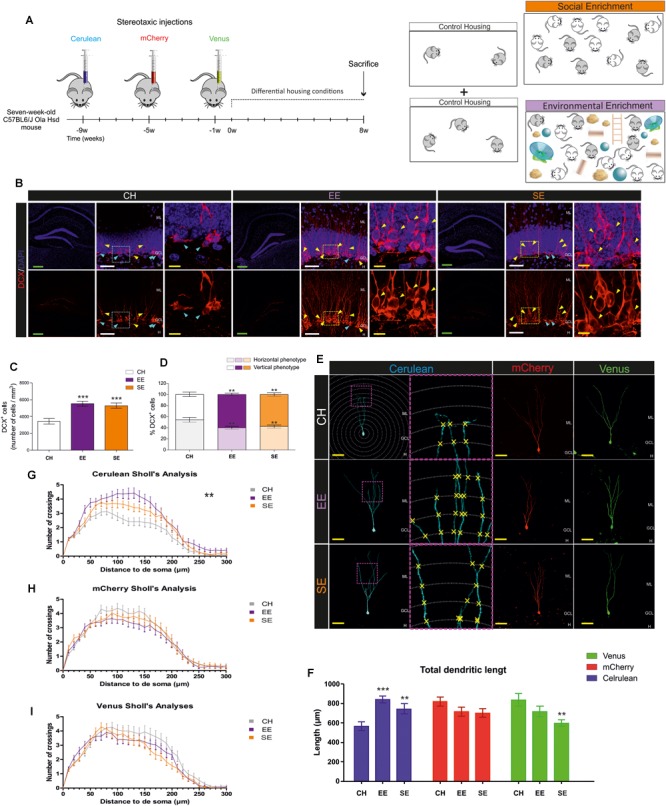
Effects of Environmental enrichment (EE) and Social enrichment (SE) on the number and morphological maturation of newborn dentate granule cells (DGCs). **(A)** Experimental design. **(B)** Representative images of Doublecortin-expressing (DCX^+^) cells in control housing (CH), EE, and SE animals. **(C)** Number of DCX^+^ cells. **(D)** Percentage of “Horizontal type” and “Vertical type” DCX^+^ cells in the different experimental conditions. **(E)** Representative images of newborn dentate granule cells (DGCs) transduced with either Cerulean-, mCherry-, or Venus-encoding retroviruses in the different experimental conditions. **(F)** Total dendritic length of newborn DGCs transduced with either Cerulean-, mCherry-, or Venus-encoding retroviruses in the different experimental conditions. **(G)** Sholl’s analysis of dendritic branching in Cerulean-transduced newborn DGCs. **(H)** Sholl’s analysis of dendritic branching in mCherry-transduced newborn DGCs. **(I)** Sholl’s analysis of dendritic branching in Venus-transduced newborn DGCs. ML, molecular layer; GCL, granule cell layer; H, hilus. Green scale bar: 250 μm. White scale bar: 50 μm. Yellow scale bar: 10 μm. Blue triangles: “Horizontal type” DCX^+^ cells. Yellow triangles: “Vertical type” DCX^+^ cells. ^∗∗^0.01 > *p* ≥ 0.001; ^∗∗∗^*p* < 0.001. Asterisks indicate changes with respect to CH animals.

To label three cell subpopulations of newborn DGCs of different ages in the same mouse, we stereotaxically injected each one of the three so-called *RGB retroviruses* (Gomez-Nicola et al., 2014) at a different time point. The time schedule of stereotaxic injections is shown in Figure 1A. 1 week after the last injection, animals were assigned to one of three experimental conditions, namely Control Housing (CH), EE, or SE. Mice were housed under these conditions for the following 8 weeks. It should be noted that stereotaxically injected mice were housed with naïve age-matched counterparts under each experimental condition. Consequently, each experimental group comprised five stereotaxically injected mice +7 naïve mice. After completion of this 8-week period, naïve mice were subjected to the Open Field and Social Interaction behavioral tests. Finally, the animals were sacrificed and immunohistochemical determinations were performed. Animals in EE and SE conditions were housed in groups of 12 animals per cage, whereas four mice were housed together in CH conditions.

### Preparation of Viral Stocks

We used three retroviral stocks encoding for either mCherry (Red, R), Venus (Green, G), or Cerulean (Blue, B) fluorescent proteins on a RSF91 backbone (Schambach et al., 2006; Gomez-Nicola et al., 2014). The plasmids used to produce these viruses were kindly provided by Profs. Tsien (Howard Hughes Medical Institute Laboratory at the University of California, San Diego, CA, United States), Baum and Schambach (Hannover Medical School, Germany), Miyawaki (RIKEN Brain Science Institute, Saitama, Japan), Riecken (University Medical Center Hamburg-Eppendorf, Germany), and Gage (Salk Institute, CA, United States). Retroviral stocks were concentrated to working titers of 1 × 10^7^–2 × 10^8^ pfu/ml by ultracentrifugation (Zhao et al., 2006). Since the retroviruses used are engineered to be replication-incompetent, only cells dividing at the time of surgery are infected (Zhao et al., 2006). In the dentate gyrus (DG), these proliferative cells are almost totally restricted to newborn DGCs (Zhao et al., 2006).

### Stereotaxic Surgery

Seven-week-old mice were anesthetized with isoflourane and placed in a stereotaxic frame. Viruses were injected into the DG at the following coordinates (mm) relative to bregma [-2.0, ± 1.4, 2.2] in the anteroposterior, mediolateral, and dorsoventral axes. 2 μl of each retrovirus was injected with a glass micropipette at a rate of 0.2 μl/min. Micropipettes were kept in place at the site of injection for an additional 5 min to avoid any suction effect of the solution injected, before being slowly removed.

### Differential Housing Conditions

Housing under differential conditions started 1 week after the last stereotaxic injection was performed and lasted 8 weeks.

#### Control Housing (CH)

Animals under CH conditions were housed in groups of 2–3 in standard polycarbonate cages.

#### Environmental Enrichment (EE)

We used a previously described EE protocol (Llorens-Martin et al., 2010). All enriched cages were equipped with various types of running wheel. Animals under EE conditions were housed in groups of 12 (five stereotaxically injected +7 naïve animals) in large transparent polycarbonate cages (55 cm × 33 cm × 20 cm, Plexx Ref. 13005). They had free access to voluntary running wheels and toys of different shapes, sizes, materials, and surface textures. A set of 10 different toys and new bedding were placed in the cages every other day in order to alter the environment (Llorens-Martin et al., 2010).

#### Social Enrichment (SE)

Animals under SE conditions were housed in groups of 12 (five stereotaxically injected +7 naïve animals) in large transparent polycarbonate cages (55 cm × 33 cm × 20 cm, Plexx Ref. 13005).

### Sacrifice

Mice were fully anesthetized by an intraperitoneal injection of pentobarbital (EutaLender, 60 mg/kg) and transcardially perfused with 0.9% saline followed by 4% paraformaldehyde in 0.1 N phosphate buffer (PB). Brains were immediately removed and post-fixed at 4°C overnight in the same fixative. They were then washed three times in 0.1 N PB.

### Immunohistochemistry (IHC)

We obtained 50-μm thick coronal brain sections on a Leica VT1200S vibratome (Vivar et al., 2012). Series of brain slices were randomly made up of one section from every eighth for immunohistochemical analyses. Slices were initially pre-incubated in phosphate buffer with 1% Triton X-100 and 1% bovine serum albumin for 10 min. Dual or triple immunohistochemistry was then performed as described previously (Llorens-Martin et al., 2013), using the following primary antibodies: goat anti-Doublecortin (DCX) (Santa Cruz Biotechnology Cat# sc-8066, **RRID:AB_2088494**; 1:500); guinea pig anti-PSD95 (Synaptic Systems Cat# 124 014, **RRID:AB_2619800**; 1:1000); and rabbit anti-Piccolo (Synaptic Systems Cat# 224 003, **RRID:AB_2263066**; 1:500). The binding of these primary antibodies was detected by incubation with the following secondary antibodies: Alexa-488 donkey anti-rabbit (Molecular Probes Cat# A-21206, **RRID:AB_141708**; 1:1000); Alexa-488 donkey anti-mouse (Molecular Probes Cat# A-21202; 1:1000); Alexa-555 donkey anti-rabbit (Molecular Probes Cat# A-31572, **RRID:AB_162543**; 1:1000); Alexa-555 goat anti-ginea pig (Molecular Probes Cat# A-21435; 1:1000); Alexa-555 donkey anti-goat (Molecular Probes Cat# A-21432, **RRID:AB_141788**; 1:1000); Alexa-555 donkey anti-rat (Molecular Probes Cat# A-21434, **RRID:AB_141733**; 1:1000); Alexa-647 donkey anti-goat (Molecular Probes Cat# A-21447; 1:1000); and Alexa-647 donkey anti-rabbit (Molecular Probes Cat# A-31573; 1:1000). To label cell nuclei, all the sections were counterstained for 10 min with DAPI (Merck, 1:5000).

### Cell Counts

The number of DCX^+^ cells was determined using the physical dissector method coupled to confocal microscopy, as previously described (Llorens-Martin et al., 2014, 2016). Briefly, *z*-stacks of images were obtained under a LSM710 Zeiss confocal microscope (25× Oil immersion objective). Various parameters were used to accurately determine the number of DCX^+^ cells (XY dimensions: 103.81 μm; *Z*-axis interval: 1.7 μm; *Z*-axis thickness: 20 μm). Five stacks of 20 images were analyzed per cell marker and animal. The number of cells counted was divided by the reference volume in order to calculate cell densities, as previously described (Llorens-Martin et al., 2013).

Moreover, to determine the effects of EE on the morphological maturation of DCX^+^ cells, these cells were classified following previously published criteria (Plumpe et al., 2006). In this regard, “Horizontal type” referred to DCX^+^ cells with an immature morphology, which included those with no neurites or those with several undifferentiated neurites oriented parallel to the hilar border of the granule cell layer. In contrast, “Vertical type” referred to more differentiated DCX^+^ cells that exhibited a single primary apical neurite oriented perpendicular to the hilar border toward the molecular layer. We calculated the percentage of cells of each type and used averaged values per animal in the graphs (Figure 1D).

### Morphometric Analysis of Retrovirally Labeled Newborn Dentate Granule Cells

RGB retroviruses have traditionally been injected simultaneously as a cocktail (Gomez-Nicola et al., 2014). However, in order to label three cell populations of different ages in the same animal, each retrovirus was injected at a different time point. This experimental design allowed the labeling of newborn DGCs in three colors, namely red (mCherry^+^), green (Venus^+^), and blue (Cerulean^+^) (Schambach et al., 2006; Gomez-Nicola et al., 2014). At least 50 randomly selected newborn DGCs per mouse were reconstructed in a LSM710 Zeiss confocal microscope (25× oil immersion objective, XY dimensions: 103.81 μm). Confocal stacks of images were obtained (*Z*-axis interval: 2 μm), and *Z*-projections were analyzed. Total dendritic length was determined using the *NeuronJ* plugin in *Fiji*. Dendritic branching was analyzed using the *ShollAnalysis* plugin for *Fiji* (Llorens-Martin et al., 2013; Pallas-Bazarra et al., 2017).

### Measurement of PSD95^+^ and Piccolo^+^ Area

Five confocal images corresponding to each of the three sub-regions of the molecular layer (ML) [namely external (EML), medial (MML) and inner (IML) layer, or to the granule cell layer (GCL)] were obtained per animal in a LSM710 Zeiss confocal microscope (63× oil immersion objective; XY dimensions: 24.1 μm). Next, an invariant threshold for fluorescence intensity was established in *Fiji* software, and the area over the threshold was measured following previously described procedures (Pallas-Bazarra et al., 2016).

### Western Blotting

Extracts for western blot analysis were prepared by homogenizing the hippocampus in ice-cold extraction buffer consisting of 50 mM Tris HCl, pH 7.4, 150 mM NaCl, 1% NP-40, 1 mM sodium orthovanadate, 1 mM EDTA, a protease inhibitor cocktail (Roche), and 1 μM okadaic acid. Samples were homogenized and protein content was determined by the Pierce BCA Protein Assay (Thermo Fisher #23225) method. Twenty-five micrograms of total protein were electrophoresed on 10% SDS-polyacrylamide gel and transferred to a nitrocellulose membrane (Schleicher & Schuell, Keene, NH, United States). Data were normalized with respect to CH mice. A mouse anti-PSD95 (UC Davis/NIH NeuroMab Facility Cat# 75-028, **RRID: AB_2292909**; 1:1000) primary antibody was used. Membranes were incubated with the antibody at 4°C overnight in 5% nonfat dried milk. Secondary anti-Mouse antibodies (1:1000; Invitrogen, San Diego, CA, United States) were incubated for 2 h at room temperature and ECL detection reagents (Amersham Biosciences, Arlington Heights, IL, United States) were used for immunodetection. Quantification was performed by densitometric scanning. The densitometry values were obtained in the linear range of detection with these antibodies. Values were normalized with respect to anti-β-Actin (Sigma Cat#A5441, **RRID: AB_476744**; 1:5000) to correct for total protein content.

### Behavioral Tests

#### Open Field

**FIGURE 2:**
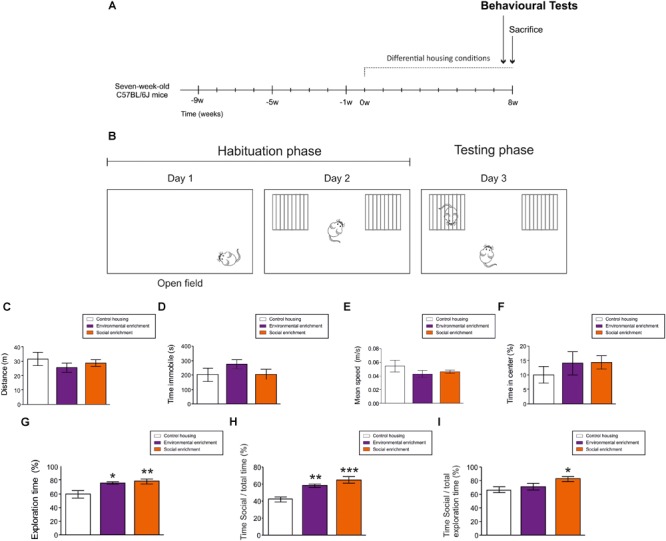
Behavioral effects of Environmental enrichment (EE) and Social enrichment (SE). **(A)** Experimental design. **(B)** Schematic diagram of behavioral test performed. **(C)** Total distance moved in Open field test. **(D)** Total time immobile in Open field test. **(E)** Mean speed in Open field test. **(F)** Percentage of time spent in the central part of the arena in Open field test. **(G)** Total exploration time during day 2. **(H)** Percentage of time exploring the cage holding a mouse divided by the total testing time during day 3. **(I)** Percentage of time exploring the cage holding a mouse divided by the total exploratory time during day 3. ^∗^0.05 > *p* ≥ 0.01; ^∗∗^0.01 > *p* ≥ 0.001; ^∗∗∗^*p* < 0.001. Asterisks indicate changes with respect to control housing animals.

To analyze general ambulatory and anxiety-like behaviors, animals were exposed to a square (45 cm × 45 cm), constantly illuminated, open-field methacrylate arena for 10 min (See Figure 2A,B for schematic diagrams). The behavior of the animals was recorded with a zenithal video camera connected to AnyMaze (Stoelting Co.) software. The distance and speed traveled, the percentage of time the animals spent immobile, and the percentage spent in the center of the arena were calculated using the software (Llorens-Martin et al., 2014).

#### Social Interaction Test

##### Habituation phase

24 h after being tested in the Open field, mice were placed in the same cage for 10 min. Cages were equipped with two empty identical compartments made of thin iron bars. The percentage of time the animals spent exploring these compartments was calculated.

##### Testing phase

24 h later, animals were exposed to the same environment explored during the training phase. In this case, one of the compartments held a non-familiar female mouse. The percentage of time the animals spent interacting with this mouse (either relative to the total exploratory or the total testing time) was calculated as a measurement of social interaction (McQuaid et al., 2018).

### Statistical Analysis

Statistical analysis was performed using SPSS 25 software (SPSS, 1989; Apache Software Foundation, Chicago, IL, United States). To test the normality of sample distribution, the Kolmogorov–Smirnov test was used. For comparisons between the three experimental groups, a One-way ANOVA test was used in case of normal sample distribution, whereas the nonparametric Kruskal-Wallis test was used in those cases in which normality could not be assumed. The DMS *post hoc* test was used to compare individual differences between groups when the aforementioned tests produced statistically significant differences. Dendritic arborization (Sholl’s analysis) was analyzed using a repeated measurements ANOVA test. Simple main effects of genotype at each point of the dendritic tree were tested to determine the differences between the groups. Graphs represent mean values ±SEM. A 95% confidence interval was used for statistical comparisons.

## Results

### Effects of Social Enrichment and Environmental Enrichment on the Number and Morphological Maturation of Newborn Dentate Granule Cells

Mice were housed under CH, SE, or EE conditions for 8 weeks. To label cellular populations of three distinct ages, animals received single stereotaxic injections of a retrovirus that encodes one of the following fluorescent proteins: Cerulean, mCherry, or Venus. Stereotaxic injections were performed at different time points following the experimental design shown in Figure 1A. In addition, seven additional mice per housing condition did not receive any stereotaxic injection but were housed under the same conditions. These animals were used to perform cellular counts and behavioral analyses.

First, we counted the number of Doublecortin-positive (DCX^+^) cells in the different experimental conditions. Both EE (*p* ≤ 0.001) and SE (*p* = 0.008) produced a similar increase in the number of these cells with respect to CH conditions (*F*_2,4_ = 10.419; *p* ≤ 0.001). No differences were observed in this parameter between SE and EE mice (*p* = 0.993). In Figure 1B, representative images of DCX^+^ cells are shown. High-power magnification images show two populations of DCX^+^ cells with remarkable morphological differences. As can be observed, some cells exhibited an immature phenotype (characterized by the presence of several neurites oriented parallel to the SGZ), whereas others had a more mature phenotype (characterized by the presence of a single primary apical dendrite perpendicular to the SGZ and oriented toward the ML) (Figure 1C). EE and SE conditions increased the morphological maturation of these cells, since both (*p* = 0.002 and *p* = 0.009, respectively) increased the percentage of the more differentiated DCX^+^ cells, and reduced that of the immature type cells (*F*_2,4_ = 5.755; *p* = 0.004) (Figure 1D). No differences in the percentage of immature (*p* = 0.637) or mature (*p* = 0.637) DCX^+^ cells were observed between SE and EE mice.

Next, we analyzed the effects of housing conditions on the morphology of previously generated newborn DGCs, which were 9- (Cerulean^+^), 5- (mCherry^+^), or 1- (Venus^+^) week-old at the beginning of the differential housing conditions (Figure 1E). EE (*p* ≤ 0.001) and SE (*p* ≤ 0.001) increased the total dendritic length of 17-week-old Cerulean^+^ newborn DGCs with respect to CH conditions (*F*_2,4_ = 10.307; *p* ≤ 0.001) (Figure 1F). Accordingly, EE (*p* ≤ 0.001) and SE (*p* = 0.033) increased the dendritic branching of these cells (Greenhouse-Geisser Interaction *F* = 2.600; *p* = 0.008) (Figure 1G). No differences in the total dendritic length (*p* = 0.130) of Cerulean^+^ cells were observed between SE and EE mice, although differences in dendritic branching were detected (*p* = 0.017). In contrast, none of these conditions modified the total dendritic length (*F*_2,4_ = 2.044; *p* = 0.136) (Figure 1F) or dendritic branching [Greenhouse-Geisser Interaction *F* = 0.903; *p* = 0.475) (Figure 1H) of 13-week-old mCherry^+^ cells. Finally, SE (*p* = 0.003 (with respect to WT mice), and *p* = 0.126 (with respect to EE mice)] caused a slight decrease in the total dendritic length of 9-week-old Venus^+^ newborn DGCs (*F*_2,4_ = 4.743; *p* = 0.011) (Figure 1F), although the branching pattern remained unaltered in these cells (Greenhouse-Geisser Interaction *F* = 1.295; *p* = 0.269) (Figure 1I).

These data support the notion that SE triggers effects similar to those induced by EE on the morphological maturation of newborn DGCs of different ages.

### Behavioral Effects of Social Enrichment and Environmental Enrichment

To test the potential effects of EE and SE on ambulatory, anxiety-like and exploratory behaviors, animals were tested in the Open field (OF) and the Social interaction (SI) tests, respectively (Figure 2A,B). During the OF test, housing conditions did not affect the total distance moved (*F*_2,4_ = 0.711; *p* = 0.505) (Figure 2C), the immobility time (*F*_2,4_ = 1.225; *p* = 0.317) (Figure 2D), the speed of displacement (*F*_2,4_ = 0.206; *p* = 0.816) (Figure 2E), or the percentage of time spent in the center of the arena (*F*_2,4_ = 1.612; *p* = 0.229) (Figure 2F). These data suggest that housing conditions did not affect the ambulatory or anxiety-like behavior of the mice.

In contrast, both EE (*p* = 0.04) and SE (*p* = 0.010) increased the percentage of time the animals spent exploring the empty compartments during the second day (*F*_2,4_ = 6.533; *p* = 0.007). No differences in this parameter were observed between SE and EE mice (*p* = 0.670) (Figure 2G). Moreover, EE (*p* ≤ 0.001) and SE (*p* = 0.004) led to an increase in the percentage of time the mice spent exploring the compartment that held another mouse [with respect to the total testing time (*F*_2,4_ = 12.901; *p* ≤ 0.001) (Figure 2H)], although only SE (*p* = 0.016 with respect to WT mice) increased the percentage of time the animals spent exploring the cage containing another mouse [with respect to the total exploratory time (*F*_2,4_ = 3.686; *p* = 0.046) (Figure 2I)].

These data suggest that housing conditions determine the social and exploratory behaviors of female C57/BL6 mice without affecting their locomotor activity.

### Effects of Housing Conditions on the Afferent Excitatory Connections of the Dentate Gyrus

To test the potential effect of housing conditions on the excitatory afferent connections to the DG, we analyzed the expression of a presynaptic (namely, Piccolo) and a postsynaptic (namely, PSD95) marker of glutamatergic synapses in several regions of the ML and in the GCL (Supplementary Figures S1A–C). To this end, the number and area occupied by PSD95^+^ and Piccolo^+^ particles were determined. The number of PSD95^+^ puncta (Supplementary Figure S1B) was increased by EE (*p* = 0.006) in the IML (*F*_2,4_ = 5.208; *p* = 0.008). Accordingly, the area occupied by these particles was also increased by EE in the same region (*F*_2,4_ = 15.209; *p* ≤ 0.001) (Supplementary Figure S1C). Regarding the expression of Piccolo, EE increased the number of these particles in the EML (*F*_2,4_ = 7.874; *p* = 0.001), MML (*F*_2,4_ = 6.775; *p* = 0.002), and IML (*F*_2,4_ = 4.027; *p* = 0.023) regions of the ML, and in the GCL (*F*_2,4_ = 4.806; *p* = 0.011) (Supplementary Figure S1C). Accordingly, the area occupied by these particles was also increased in the same regions (EML: *F*_2,4_ = 23.740; *p* ≤ 0.001; MML: *F*_2,4_ = 24.268; *p* ≤ 0.001; IML: *F*_2,4_ = 31.642; *p* ≤ 0.001; and GCL: *F*_2,4_ = 5.555; *p* = 0.006) (Supplementary Figure S1E). However, no changes in markers of excitatory synapses were observed in SE animals. These data suggest that EE and SE exert differential effects on the afferent connectivity of the DG.

Moreover, we measured the protein levels of PSD95 in the hippocampus of CH, EE, and SE mice (Supplementary Figures S1F,G). As shown, no differences in the levels of PSD95 (*F*_2,4_ = 2.752; *p* = 0.083) expression were observed between the different experimental groups. These data point to a selective increase in the expression of excitatory synapse markers in the ML caused by EE.

## Discussion

The rodent DG continuously gives rise to new DGCs during life (Altman and Das, 1965). Newborn DGCs go through a tightly orchestrated sequence of maturative stages before becoming fully integrated into the pre-existing hippocampal trisynaptic circuit (Zhao et al., 2006). In this regard, EE and physical exercise are two of the most potent positive modulators of the rate of AHN (Kempermann et al., 1997; van Praag et al., 1999). The pro-neurogenic effects of EE are complex and multi-faceted since this manipulation differentially affects the sequential maturative stages that newborn DGCs go through. In this regard, EE increases the survival and synaptic integration of newly generated DGCs (van Praag et al., 2000) and exerts a profound rewiring of their afferent connectivity (Bergami et al., 2015). However, recent evidence points to remarkable differences in the maturation-promoting effects of EE on newborn DGCs of different ages. In fact, a recent concept, referred to as the *critical period*, reflects the limited period during which newborn DGCs exhibit the highest sensitivity to the stimulatory effects of EE and physical exercise (Bergami et al., 2015; Temprana et al., 2015). However, the duration of this period remains controversial. A study by Bergami et al. (2015) showed that a 4-week period of EE selectively increases the maturation and number of afferent synaptic partners of 2- to 6-week-old DGCs. In contrast, Alvarez et al. demonstrated that newborn DGCs exhibit the highest sensitivity to the stimulatory effects of a 2-day period of EE between the first and the second week of cell age (Alvarez et al., 2016). Moreover, studies by Sah et al. (2017) and Vivar et al. (2016) showed that running rewires the afferent connections of 1- and 4-week-old newborn DGCs respectively. Previous data from our laboratory indicate that a 6- to 8-week period of EE increases the number of postsynaptic densities and the size of mossy fiber terminals (MFTs) of 4- and 1-week-old newborn DGCs, respectively (Llorens-Martin et al., 2013; Pallas-Bazarra et al., 2016). Moreover, other authors demonstrate that EE increases the morphological complexity of fully mature DGCs (Faherty et al., 2003; Darmopil et al., 2009), although it does not modify this parameter in 4-week-old newborn DGCs (Llorens-Martin et al., 2013). As shown, research conducted by various groups point to differential, or even contradictory, effects of EE on the maturation of newborn DGCs of distinct ages. In the present study, we aimed to address these differences by labeling three populations of newborn DGCs in the same mouse. Our data show that under a paradigm in which animals received three subsequent stereotaxic injections, only 9-week-old cells exhibited an increase in dendritic branching and length after EE, whereas 4- and 1-week-old newborn DGCs did not experience variations in these parameters in response to an 8-week period of EE. Despite the prolonged inter-stereotaxic injection interval, the possibility that the inflammation caused by previous injections conditioned the morphological development of mCherry^+^ and Venus^+^ cells in response to enriched conditions cannot be completely ruled out. However, the number and morphological maturation of DCX^+^ cells born during the course of the EE period was markedly increased by enriched conditions, which strongly argues against the aforementioned possibility. Interestingly, similar effects on the population of DCX^+^ cells have been reported after both short- (Beauquis et al., 2010) and long-term EE protocols (Llorens-Martin et al., 2007). Thus, our data confirms that newborn DGCs of different ages show a markedly different sensitivity to the stimulatory actions of EE.

Regarding the pro-neurogenic mechanisms triggered by EE, various components of this paradigm have been demonstrated to potentiate specific stages of the neurogenic process (Fabel et al., 2009). It has been largely acknowledged that the increased physical activity component of EE selectively increases precursor cell proliferation (van Praag et al., 1999; Holmes et al., 2004; Plumpe et al., 2006; Fabel and Kempermann, 2008; Steiner et al., 2008; Kobilo et al., 2011; Marlatt et al., 2012), whereas exercise is not required to enhance the survival of these cells (Brown et al., 2003; Olson et al., 2006; Fabel et al., 2009). Among the exercise-independent components of the EE paradigm, one critical aspect, namely the increased social interaction between enriched mice, has received little attention in the literature. Although long-lasting pro-neurogenic effects of an early post-weaning model of SE, referred to as communal nesting, have been reported (Branchi et al., 2006b), the putative existence of these effects when SE is applied to mice during adulthood have not been explored to date. To address this question, we compared the pro-neurogenic effects of an 8-week period of SE to those of a standard protocol of EE and to CH conditions. Surprisingly, we found that SE increased the number of DCX^+^ cells and the morphological maturation of newborn DGCs of different ages, and these effects were comparable to those exerted by EE. It should be noted that our study included mice housed in small groups as in CH conditions, rather than socially isolated animals, the latter being a highly stressful situation for social rodents with detrimental effects on the rate of AHN (Stranahan et al., 2006; Ibi et al., 2008; Kulesskaya et al., 2011). Thus, our data reveal unexpected effects of SE on newborn DGCs of different ages. This manipulation increased the number and morphological maturation of DCX^+^ cells and selectively increased the dendritic length and branching of 9-week-old retrovirally labeled newborn DGCs. Importantly, the magnitude of these effects was equal to that observed in EE mice. Hence, our findings shed light on a crucial component of EE, namely increased social interaction, as a potent stimulator of AHN in the absence of any further cognitive stimulation or increased physical activity.

Furthermore, EE exerts a plethora of neurogenesis-dependent and independent effects on hippocampal plasticity and behavior (Farmer et al., 2004; Meshi et al., 2006; Pena et al., 2006; Segovia et al., 2006; Schloesser et al., 2010; Bekinschtein et al., 2011; Kulesskaya et al., 2011; Lehmann and Herkenham, 2011; Doulames et al., 2014; Freund et al., 2015; Bechard et al., 2016; Garthe et al., 2016). Regarding the behavioral consequences of SE, they have been reported to be strongly dependent on species, gender and the developmental period at which the intervention is applied. In general terms, early post-weaning SE increases stress resilience (Cirulli et al., 2010; Branchi et al., 2013) and alters anxiety-like or depressive-like behaviors (Branchi and Alleva, 2006; Branchi et al., 2006b; Curley et al., 2009; D’Andrea et al., 2010), whereas when applied during adulthood it either increases (Branchi et al., 2006a) or decreases (Gracceva et al., 2009) social interaction. Here we observed no alteration of motor behavior in any of the experimental groups studied. However, we detected a selective increase in social interaction in response to SE and EE. In this regard, previous observations reported that EE increased the number of social interaction contacts between adult female mice (Freund et al., 2015). This finding, together with the observation that EE and SE regulated excitatory afferent innervation in the DG in distinct manners, points to differential mechanisms of action exerted by the components of EE.

In addition to the aforementioned effects of EE and SE under physiological conditions, both interventions have been demonstrated to have therapeutic effects under pathological conditions. In this regard, both EE and SE induce recovery in various models of central nervous system injury (Berrocal et al., 2007; Gajhede Gram et al., 2015; Lajud et al., 2018). Moreover, EE increases AHN in mouse models of Down syndrome (Chakrabarti et al., 2011; Pons-Espinal et al., 2013), Alzheimer’s disease (Levi and Michaelson, 2007; Mirochnic et al., 2009; Rodriguez et al., 2011; Valero et al., 2011; Llorens-Martin et al., 2013; Marlatt et al., 2013), Huntington’s disease (Lazic et al., 2006), diabetes (Pamidi and Nayak, 2014), ischemia (Rojas et al., 2013), and chronic pain (Zheng et al., 2017), after cranial irradiation (Garbugino et al., 2016), and during physiological aging (Kempermann et al., 1998, 2002; Kempermann, 2008, 2015; Speisman et al., 2013). In contrast, early SE reverses social deficits in animal models of autism (Garbugino et al., 2016; Campolongo et al., 2018), Parkinson’s disease (Goldberg et al., 2012), and Fragile X syndrome (Oddi et al., 2015). Thus, our data take on greater relevance when examined from a therapeutic perspective. Given that SE alone is capable of inducing potent pro-neurogenic effects in the absence of any further cognitive stimulation or increase in physical activity and that these effects are of a similar magnitude to those exerted by EE, then SE emerges as an interesting alternative approach to increase AHN under certain pathological conditions. In this regard, it should be noted that numerous neurodegenerative conditions course with motor coordination impairments and with a general compromise of motor abilities. Hence, the possibility of increasing AHN by means of SE gains further relevance in the context of these pathological conditions.

## Ethics Statement

Five-week-old female C57BL6/J Ola Hsd mice were purchased from EnVigo laboratories (Spain). Animals were housed at the Centro de Biología Molecular “Severo Ochoa” (CBMSO) in a specific pathogen-free colony facility in accordance with European Community Guidelines (directive 86/609/EEC) and handled following European and local animal care protocols. Given that the hierarchy/dominance relationships established between male mice have a negative impact on AHN (Gracceva et al., 2009; D’Andrea et al., 2010), only female mice were used in this work. Animals were left undisturbed for 2 weeks before starting any experimental manipulation. During this period, they were housed in groups of four mice per cage. Experiments were approved by the CBMSO Ethics Committee (AEEC-CBMSO-23/172) and the National Ethics Committee (PROEX 205/15). In stereotaxic injection experiments, five mice were used for each experimental condition. In cell count and behavioral determination experiments, seven animals per experimental condition were used.

## Author Contributions

ML-M designed and conceived the experiments. EM-J, JJ-A, and ML-M performed the experiments. EM-J and ML-M acquired confocal images. EM-J analyzed the data. EM-J and ML-M wrote the manuscript. JA and ML-M obtained funding. All the authors critically discussed the data and revised the final version of the manuscript.

## Conflict of Interest Statement

The authors declare that the research was conducted in the absence of any commercial or financial relationships that could be construed as a potential conflict of interest.

## Supplementary Material

FIGURE S1 Non-neurogenic effects of Environmental enrichment (EE) and Social enrichment (SE). **(A)** Representative high-power magnification images showing staining with presynaptic (Piccolo) and postsynaptic (PSD95) markers of glutamatergic synapses in the Molecular layer (ML) of the DG. **(B)** Density of PSD95^+^ particles in various sub-regions of the DG. **(C)** Density of Piccolo^+^ particles in sub-regions of the DG. **(D)** Total PSD95^+^ area in sub-regions of the DG. **(E)** Total Piccolo^+^ area in different sub-regions of the DG. **(F)** Measurement of PSD95 protein expression level in the hippocampus by western Blot (WB). Anti-β-Actin was used as a loading control. Data are normalized to the expression levels of control housing (CH) mice. **(G)** WB membranes showing PSD95 expression in the different experimental groups. EML, external molecular layer; MML, medial molecular layer; IML, inner molecular layer; GCL, granule cell layer. Yellow scale bar: 5 μm. ^∗∗^0.01 > *p* ≥ 0.001; ^∗∗∗^*p* < 0.001. Asterisks indicate changes with respect to control housing animals.
