# Supplementary figures and images for: The Social Component of Environmental Enrichment Is a Pro-neurogenic Stimulus in Adult c57BL6 Female Mice
Source: Front Cell Dev Biol. 2019 Apr 26;7:62. doi: 10.3389/fcell.2019.00062 (PMC6497743; doi:10.3389/fcell.2019.00062)

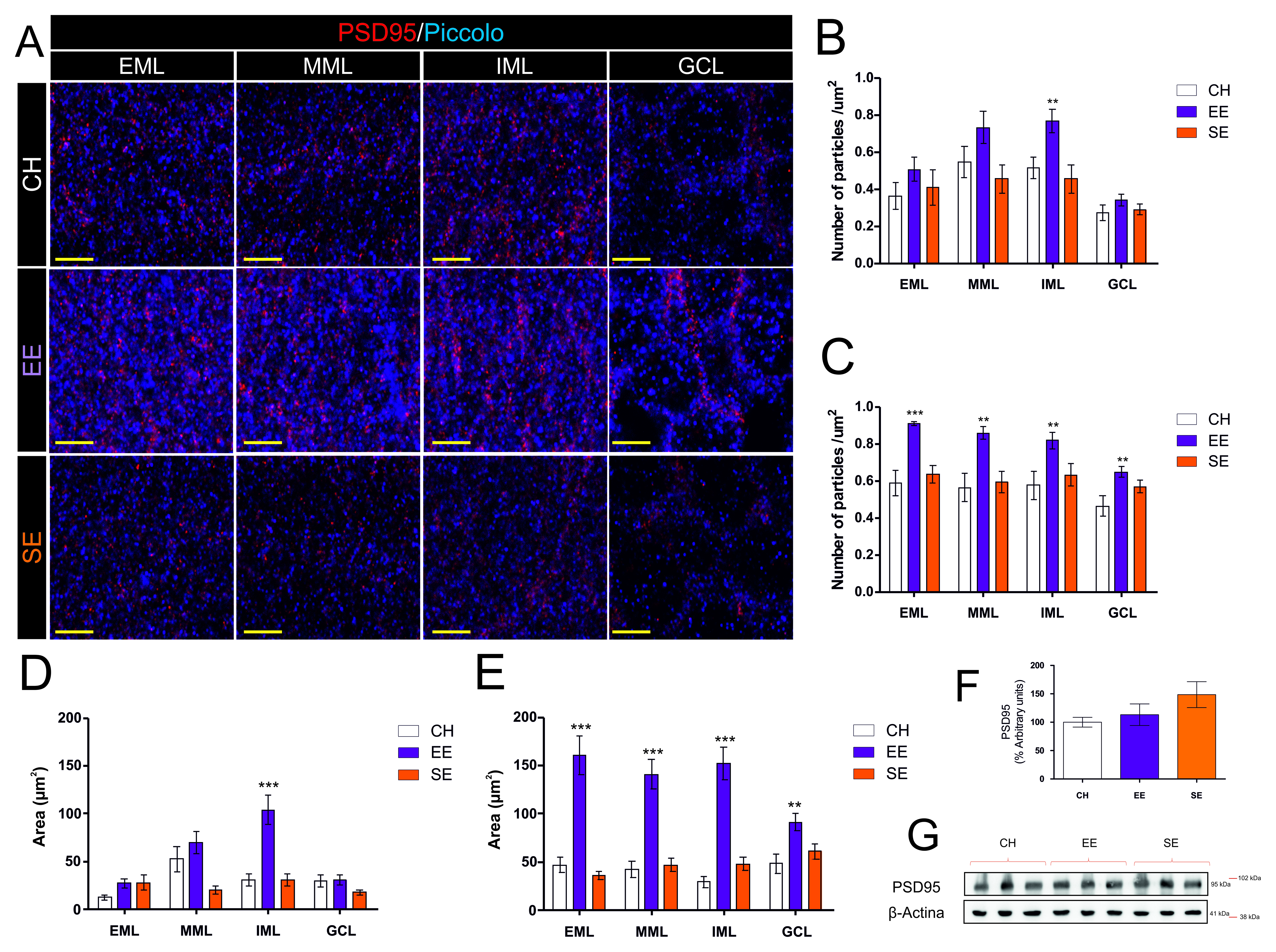

Supplement: FIGURE S1 — Non-neurogenic effects of Environmental enrichment (EE) and Social enrichment (SE). (A) Representative high-power magnification images showing staining with presynaptic (Piccolo) and postsynaptic (PSD95) markers of glutamatergic synapses in the Molecular layer (ML) of the DG. (B) Density of PSD95+ particles in various sub-regions of the DG. (C) Density of Piccolo+ particles in sub-regions of the DG. (D) Total PSD95+ area in sub-regions of the DG. (E) Total Piccolo+ area in different sub-regions of the DG. (F) Measurement of PSD95 protein expression level in the hippocampus by western Blot (WB). Anti-β-Actin was used as a loading control. Data are normalized to the expression levels of control housing (CH) mice. (G) WB membranes showing PSD95 expression in the different experimental groups. EML, external molecular layer; MML, medial molecular layer; IML, inner molecular layer; GCL, granule cell layer. Yellow scale bar: 5 μm. ∗∗0.01 > p ≥ 0.001; ∗∗∗p < 0.001. Asterisks indicate changes with respect to control housing animals. [file Image_1.TIF]
